# Personal protection of long lasting insecticide-treated nets in areas of *Anopheles gambiae *s.s. resistance to pyrethroids

**DOI:** 10.1186/1475-2875-5-12

**Published:** 2006-02-10

**Authors:** Roch K Dabiré, Abdoulaye Diabaté, Thierry Baldet, Léa Paré-Toé, Robert T Guiguemdé, Jean-Bosco Ouédraogo, Ole Skovmand

**Affiliations:** 1 IRSS, Direction régionale de Bobo-Dioulasso, BP 545 Bobo-Dioulasso, Burkina Faso, France; 2 Centre Muraz, BP 390 Bobo-Dioulasso, Burkina Faso, France; 3 CIRAD-EMVT, Campus International de Baillarguet 34398 Montpellier Cedex 5, France; 4 Intelligent Insect Control, Montpellier, France

## Abstract

**Background:**

The development of mosquito nets pre-treated with insecticide, Long Lasting Impregnated Nets (LLINs) that last the life span of the net, is a solution to the difficulty of the re-impregnation of conventional nets. Even if they showed a good efficacy in control conditions, their efficacy in the field, particularly in areas with resistance of *Anopheles gambiae *to pyrethroids, is not well documented. This study compares wide (Olyset^®^) and small (Permanet^®^) mesh LLINs in field conditions, using entomological parameters.

**Methods:**

The two LLINs were tested in a rice-growing area of south-western Burkina Faso (West Africa) with year around high density of the main malaria vector *An. gambiae s.s*. In the study village (VK6), there is a mixed population of two molecular forms of *An. gambiae*, the S-form which dominates during the rainy season and the M-form which dominates the rest of the year. The two LLINs Olyset^® ^and Permanet^® ^were distributed in the village and 20 matched houses were selected for comparison with four houses without treated nets.

**Results:**

Mosquito entrance rate was ten fold higher in control houses than in houses with LLINs and there was no difference between the two net types. Among mosquitoes found in the houses, 36 % were dead in LLIN houses compared to 0% in control houses. Blood feeding rate was 80 % in control houses compared to 43 % in LLIN houses. The type of net did not significantly impact any of these parameters. No mosquitoes were found inside Permanet^®^, whereas dead or dying mosquitoes were collected inside the Olyset^®^. More than 60% of mosquitoes found on top or inside the nets had had blood meals from cattle, as shown by ELISA analysis.

**Conclusion:**

The percentage of blood-fed mosquitoes in a bed net study does not necessarily determine net success. The efficacy of the two types of LLINs was comparable, during a period when the S-form of *An. gambiae *was carrying the *kdr *gene. Significantly higher numbers of mosquitoes were collected in control houses compared to intervention houses, indicating that the LLINs provided an additional deterrent effect, which enhanced their expected prevention capacity.

## Background

The use of insecticide-treated bed nets (ITNs) for individual as for collective protection against malaria has been shown to reduce morbidity of childhood malaria (below five years of age) by 50% and global child mortality by 20%–30% [1-3]. ITNs are now considered to represent efficient tools for malaria vector control, when used on a large scale [4,5]. One of the key issues for their use on a large scale is the impregnation and the re-impregnation that needs technical skills and materials, which may not always be available [6]. Preliminary surveys have shown that less than 5% of nets available in Africa were properly treated or re-treated. The use of mosquito nets pre-treated with insecticide, Long Lasting Impregnated Nets (LLINs), that last the life span of the net, is a solution to this problem [7]. Two LLINs are now available and have been preliminarly recommended by WHO for malaria prevention: the Olyset^® ^[8] net, made of polyethylene netting material (mesh 20 holes/cm^2^) with permethrin (2% of concentration) incorporated into the polymer before monofilament yarn extrusion, and the Permanet^® ^[9] net, made of polyester netting material (mesh 25 holes/cm^2^) with deltamethrin incorporated (55 mg ai/m^2^) in a resin coating of the fibers.

Resistance to the insecticides used for impregnation may be a limiting factor to impregnated nets in vector control. Pyrethroid resistance of the most important African malaria vector *Anopheles gambiae *s.s. is already widespread in several West African countries [10,11]. A common resistance is caused by the *kdr *mutation that occasionally is found at very high frequency (>90%) [12,13]. Fortunately, the predominant kdr mechanism apparently does not prevent the efficacy of pyrethroid-treated bed nets [14,15]. Contrary to West Africa, malaria vector control failure due to metabolic-based resistance on pyrethroid efficacy was reported in South Africa. This resistance is closely associated with the presence of a high level of oxidase activity and sometimes conferring cross-resistance to the carbamate insecticide in the local vector *Anopheles funestus*, as recently mentioned in Kwazulu Natal and Mozambique (16-17). This oxydase-based resistance has been also observed in *An. gambiae *populations from Cameroon [18,19] and Kenya [20]. Bed nets without insecticide can never provide complete protection against blood-questing mosquitoes. However, when treated with pyrethroids, they reduce the number of mosquitoes entering houses and further reduce the blood-feeding rates of those entering. Torn impregnated nets have been shown still to reduce host/vector contact, and it has been argued that the most important effect of ITN is the mass effect on mosquitoes, whether it is before or after biting [3,21]. However, some authors considered that to be most effective, impregnated nets should have no holes, no entry flaps and should be tucked in under a mattress, which will not allow mosquitoes access inside the bed net [22]. It is likely that most people primarily acquire and use bed nets for their individual protection against mosquitoes biting [23,24].

It has been shown that mosquitoes with kdr resistance are less susceptible to the excito-repellent effect of pyrethroids [25] and it may, therefore, be possible that such mosquitoes will enter the wider meshed Olyset^® ^net and bite even when this net is still intact. Therefore, in contrast to most published studies, this study compares intact LLINs with wide (Olyset^®^) and small (Permanet^®^) mesh on the entomological parameters in an area with kdr-*An. gambiae *populations.

## Materials and methods

### Study area

**Figure 1:**
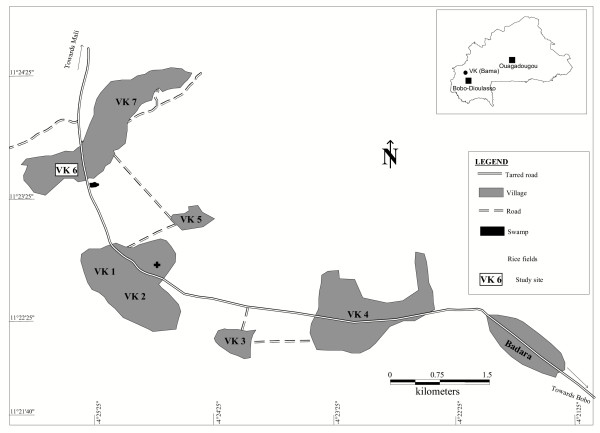
Location of the study site.

The survey was conducted in "Vallée du Kou", a rice growing area of southern Burkina Faso, West Africa. It is located at 30 km in the North of Bobo-Dioulasso between 4° 24' 42" longitude west and 11° 23' 14" latitude north and is composed of 7 villages with a total of 4,470 habitants (Figure 1). Irrigation exists in this area since 1972, and is now sub-permanent with two crops grown per year: from February to June during the dry season and from July to November during the rainy season. An unprotected human sleeping in the central rice-field area (VK5) is exposed to more than 60,000 mosquito bites/year [30]. *An. gambiae *and *An. funestus *are the two major vectors present. *Anopheles arabiensis *is rare and found in sympatry with *An. gambiae *in the surrounding savannah. The two molecular forms M and S of *An*. *gambiae *were observed in the periphery of the rice field during the rainy season. Huge amount of insecticides, mainly pyrethroids, are used to protect cotton from pest attacks in the neighbouring savannah. Previous studies conducted in this area found resistant *An. gambiae *populations to permethrin and deltamethrin in the periphery of the rice fields, but susceptible in the centre. This resistance is due to the kdr mutation, which occurs in the *An. gambiae *S-form population (95%), particularly at the end of the rainy season [11]. The kdr mutation was also observed in the M form in a very low proportion (4%) [13]. For these reasons and especially because of the high vector density throughout the year, the village of VK6 was selected to study the personal protection by Permanet^® ^and Olyset^® ^nets.

### Entomological survey

#### Mosquitoes collection

In total, 20 households matched in size and near the periphery of the rice field were equipped with Permanet^® ^and Olyset^® ^nets. Matched houses with either a Permanet^® ^or an Olyset^® ^net were not more than 50 metres apart. Mosquitoes were collected four days/month from September to November 2003. Volunteer, informed-sleepers caught blood-seeking mosquitoes in the nets from 8:00 pm to 12:00 pm and again in the early morning at 6:00 am. Residual resting indoor fauna was collected in the houses early in the morning by manual aspirators. All nets were inspected to ensure that they had no holes, no entry flaps and were tucked in under the beds. All mosquitoes found in the nets, on the nets or indoor resting or dead on the floor were collected, counted and morphologically identified. Mosquitoes were classified according to physiological status (unfed, fed and gravid). Mosquitoes collection in the morning at 6:00 am was performed in four houses without any insecticide-treated objects or nets as control. In these control houses, from 6:00 pm to 12:00 pm, blood- seeking females were caught as above and volunteers slept after 12:00 pm under their own untreated nets usually used for personal protection.

### Bloodmeal identification

The origin of bloodmeals from fed females captured above and inside the nets were identified as human or bovine using an enzyme-linked immunosorbent assay (ELISA), as described by Beier et al. [26]. In total, 45 fed-mosquitoes, respectively 24 and 21 from Olyset^® ^and Permanet^®^, representing exclusively malaria vectors were analysed by ELISA.

### Species and molecular forms of *An. gambiae *identification and kdr mutation distribution

A sample of 44 *An. gambiae *collected above and inside the nets (half from both Olyset^® ^and Permanet^®^) was selected for PCR test. Genomic DNA was extracted from single mosquitoes and PCR amplified to identify the species within *An. gambiae *complex [27], to determine the molecular form (M or S) within the species *An. gambiae *[28], and, finally, to detect the kdr mutation [29].

### Statistical analyses

Four parameters were compared between the two LLINs: (i) the percentage of house entering (compared to entrance in control houses), (ii) the number of mosquitoes collected above and inside the nets, (iii) the fed rate and (iv) the mortality rate. For each entomological parameter, comparison between treatment was made by performing a two way analysis of variance (ANOVA) and a chi square test at 95 % confidence interval.

## Results

### Mosquito house entering rate and mosquito house location

#### Global house entering rate

In total 10,621 mosquitoes were collected during the study (Table 1) and consisted mainly of *Anopheles *and *Culex *with unfed, fed, gravid, half gravid, alive or dead mosquitoes from both control and LLINs-equipped houses. 9,078 mosquitoes were caught in control houses by morning capture, while only 1,543 mosquitoes were collected in either Olyset^® ^or Permanet^® ^LLINs-equipped houses during night and in morning capture. The total number of mosquitoes collected from the different LLINs did not differ significantly (*P*>0.05). The deterrency rate of the two LLINs was similar, averaging respectively 92% and 91% in Olyset^® ^and Permanet^® ^equipped houses. The global deterrent effect, calculated as the difference of entering rates between control and total of LLINs-equipped houses, was estimated to 83% (Table 1). Globally, *Anopheles *were significantly more numerous than *Culex quinquefasciatus *irrespective of the LLINs (P = 0.01). Whatever the LLINs, *An. gambiae *was the most frequent mosquito, reaching 82%, followed by *C. quinquefasciatus *with 12% and *An. funestus *with 6.2%.

**Table 1:** Global house entering rate by mosquitoes (percentages are noted in italic) [In each line or row, values sharing a superscript letter are significantly different at 95 % confidence interval]

Mosquito species	Control	LLINs houses	Type of LLINs	
			
			Olyset^®^	Permanet^®^
*An. gambiae*	8,350	1,263	633	630
	92*	81.9*	*85.8*	*78.3*
*An. funestus*	110	95	32	63
	*1.2*	6.2*	*4.3*	*7.8*
*C. quinquefasciatus*	618	185	73	112
	6.8*	12*	*9.9*	*13.9*
Entry rate	85.5*	14.5*	6.9	7.6

% Deterrency	*83*		-	-

* Significantly different *P*<0.05

#### Mosquito house location

Mosquitoes were collected dead or alive, above or inside the nets (both Permanet^® ^and Olyset^®^) and indoor elsewhere in the house (Table 2). The number of indoor resting mosquitoes did not differ significantly with the two types of LLINs (491 for Permanet^®^,506 for Olyset^®^) and this was significantly higher than the number of mosquitoes collected from the nets (above and inside) whatever the LLINs (P < 0.05). The proportion of mosquitoes found above the nets and inside the nets was similar for the two types of nets (*P > 0.05*) with respectively 33.5% and 37.1% for Olyset^® ^and Permanet^® ^equipped houses. The number of mosquitoes collected above the nets was two fold higher on Permanet^® ^than on Olyset^®^. While dead mosquitoes from Permanet^® ^were exclusively collected above the net, half of them from Olyset^® ^were collected inside the net.

**Table 2:** Comparison of the proportion of mosquitoes according to their collection place in the LLINs equipped-houses (percentages are noted in *italic*) [In each line, values sharing a superscript letter are significantly different at 95 % confidence interval

LLINs	Resting indoor			Above LLIN			Inside LLIN			Total
		
	Ag^1^	Af	Cq	Ag	Af	Cq	Ag	Af	Cq	
Olyset^®^	398	27	66	128	2	2	107	3	5	738
	53.9*	*3.7*	*8.9*	17.3*	*0.3*	*0.3*	14.5*	*0.4*	*0.7*	
										
Permanet^®^	363	41	102	267	22	10	0	0	0	805
	45.1*	*5.1*	*12.7*	33.2*	*2.7*	*1.2*	-	-	-	

*significantly different *P*<0.05 ^1^Ag : *Anopheles gambiae*, Af : *An. funestus*., Cq : *Culex quinquefasciatus*

### Mortality rate

The global mortality rate was similar in the two LLINs houses averaging 36% (Table 4). Compared to the control houses, a high percentage of mortality was recorded in houses equipped with LLINs (36.4% *vs *0%). The proportion of dead mosquitoes collected above the net was significantly higher (37.1%, n = 299) on Permanet^® ^than that on Olyset^® ^net (17.9%, n = 132). 15.6% of dead mosquitoes were recorded inside Olyset^® ^net while no mosquito was found inside Permanet^®^. No live mosquitoes were caught above or inside the nets, but a total of 17 dying mosquitoes were collected above the two LLINs. The number of dying mosquitoes was similar in the two types of nets and was trivial compared to the total of dead mosquitoes. All mosquitoes in touch with LLINs (above or inside) were dead. Few dead mosquitoes were collected indoor the LLINs houses (n = 13 *vs *3 respectively for Olyset^® ^and Permanet^® ^equipped houses). Molecular form identification performed on 44 *An. gambiae *collected above and inside the nets showed that 88.5% of *An. gambiae *tested were of molecular M form and this proportion did not differ significantly between the two LLINs. The *kdr *gene was detected only in the all S form tested in PCR (n = 5). No *An. arabiensis *was found in the PCR-analysed mosquitoes.

**Table 4:** Comparison of the rate of fed and unfed dead females following their collection place in LLINs equipped houses (percentages are noted in *italic*)

Mosquito status	Mosquito collection place	Olyset^® ^house			Permanet^® ^house			Control house		
				
		Ag	Af	Cq	Ag	Af	Cq	Ag	Af	Cq
			
Unfed	Above LLIN	100	1	1	221	17	9	-	-	-
	Inside LLIN	84	3	3	-	-	-	-	**-**	**-**
	Resting Indoor	85	8	24	74	12	38	ND	ND	ND
				
		**309**			**371**			**726**		
Fed	Above LLIN	21	1	0	38	4	1	-	-	-
	Inside LLIN	19	0	2	-		-	-	-	-
	Resting indoor	232	16	21	171	26	25	6294	ND	ND
				
		**312**			**265**			**7242**		
		***42.3***			***32.9***			79.8*		

Gravid	Above LLIN	7	0	1	8	1	0	-	-	-
	Inside LLIN	4	0	21	-	-	-	-	-	-
	Resting indoor	81	3	21	118	3	39	ND	ND	ND
				
		**117**			**168**			**1110**		

* significantly different *P*<0.05 Ag = *Anopheles gambiae*; Af = *Anopheles funestus*; Cq = *Culex quinquefasciatus *ND : undetermined

### Mosquito blood-fed status

Less than 40% of mosquitoes collected in the LLINs equipped-houses were engorged while 79.8% were recorded in control houses (Table 5), showing a reduction of the rate of fed females averaging 42%. However, the rate of fed mosquitoes was slightly superior in Olyset^® ^-equipped houses, but it did not differ significantly between the two nets (*P > 0.05*). Within the females collected above or inside the nets (n = 247 *vs *299, respectively for Olyset^® ^and Permanet^®^), about 7% were fed mosquitoes regardless of the type of net (Table 5).

**Table 5:** Bloodmeal origin of fed *Anopheles gambiae *females from LLINs equipped houses analysed by ELISA (percentages are noted in *italic*)

Source of Bloodmeal	Olyset^®^			Permanet^®^		
	
	Inside	Above	Total	Inside	Above	Total
Human	3	2	5	0	8	8
	*-*	*-*	20.8*	-	*-*	38.1*
Bovine	12	7	19	0	13	13
	-	-	*79.2*	-	-	*61.9*

*significantly different, *P *< 0.05

### Blood meals origin

Overall, 45 specimens of engorged *An. gambiae *collected in and above the nets were analysed to determine the source of the bloodmeal. Most of the engorged females were fed on cattle, respectively 62% and 79% for Permanet^® ^and Olyset^® ^(Table 6). The rate of mosquitoes fed on humans did not differ significantly (*P*>0,05) irrespective of the LLIN, reaching respectively 21% and 30% for Olyset^® ^and Permanet^®^. The rate of mosquitoes fed on cattle was high in the LLINs equipped-houses.

## Discussion

The study was carried out in a rice field area, where high densities of mosquitoes are observed throughout the year [30]. *An. gambiae *and *C. quinquefasciatus *were resistant to pyrethroids in this area and resistance of the *An. gambiae *S form is pronounced at the end of the rainy season [11]. *Kdr *resistance was also observed in a small proportion (less than 5%) of *An. gambiae *M form, conferring a susceptible status to M population. But as *An. gambiae *S form proportion (even during its maximum peak at the end of rainy season) has never gone beyond 30 %, the global pyrethroid resistance status of this area is an intermediate resistance one [13]. This intermediate resistance status could probably explain that the two LLINs showed a global efficacy and no differential efficacy level was observed.

These results were consistent with those obtained in experimental huts from the Bouaké region in Côte d'Ivoire concerning the efficacy of LLINs in *Anopheles *and *Culex *resistant areas [15]. In contrast, in a resistance area from Danané in the north of Côte d'Ivoire, where the *An. gambiae *S form was observed with 80% of *kdr*, although the permethrin pre-impregnated Olyset nets showed a good mass effect [21], their efficacy on the reduction of entomological inoculation rate decreased significantly [31]. A similar study may be performed with deltamethrin-LLINs (Permanet^®^) in a high resistance area, as they would be expected to maintain a better efficacy, as already demonstrated in the semi-natural conditions of experimental huts [14]. Nevertheless, in the intermediate pyrethroids resistance area of the present study, an important mortality was observed both with Permanet^® ^and with Olyset^® ^nets, compare to the control. It is likely that all mosquitoes which have been in contact with the nets were killed. The few dying mosquitoes that were collected above Permanet^® ^and inside Olyset^® ^nets should be considered as truly dead mosquitoes as they were unable to fly away. Furthermore, fed mosquitoes were found dead or dying above Permanet^® ^or above/inside Olyset^® ^nets. That evokes the possibility that these mosquitoes could bite sleepers and transmit malaria in case they were infected before dying especially inside the Olyset^® ^nets. To respond to this question, sleepers were asked to keep awake in the nets and to collect mosquitoes seeking blood meal from 8:00 pm to 12:00 pm. This showed that mosquitoes caught before midnight above or inside the nets were dead or dying and some of them felt through the aperture of the net. Fed as well as unfed mosquitoes were collected in this way. Two hypothesis could be addressed to explain the presence of fed females in LLINs: i) these mosquitoes have engorged first on cattle and, as it is known that *An. gambiae *bites several times in a gonotrophic cycle [32], they may seek a human host to complete their bloodmeal, even if sleepers are protected by LLINs; ii) the engorged females collected above or inside the different nets were already blood-fed on cattle and, because in this high anopheline density area *An. gambiae *is very endophilic as a result of the use of bed nets on a large scale, they come inside houses to rest after feeding, even in LLINs-equipped houses. While seeking a place to rest in these houses, they come in contact with the nets and die.

The bloodmeal identification indicated that the majority of fed females had engorged on cattle, confirming the high zoophilic rate (about 70 %) of *An. gambiae *in this particular rice-field area, where Robert et al. [33] also observed high zoophilic rate compared to that of the neighboring savannah villages. This particular zoophilic rate can be explained by i) the proximity of livestock as each household has usually a pair of cattle for rice field tillage living the same concession; ii) because of the high density of mosquitoes in this area with more than 60,000 mosquito bites/year [30], almost every household used nets (but not necessarily treated-nets) for personal protection. For these reasons, *An. gambiae *(and other species also) developed a zoophilic behaviour for host preference. Nevertheless, some human-bloodfed females were found above and inside LLINs. This relatively low proportion of human-bloodfed mosquitoes (30%) should have been engorged on the sleepers as shown in a study carried out in Tanzania [34].

A significantly (*P *< 0.02) high proportion of unfed mosquitoes was collected in houses equipped with treated nets compared to control houses. This result confirms that LLINs clearly reduced blood meal rate as had been demonstrated in previous studies on ITNs efficacy carried out in East Africa [24,34,35].

## Conclusion

To conclude, both Permanet^® ^and Olyset^® ^showed a good efficacy against *An. gambiae *as no live individual was caught above and inside the nets. Moreover, significantly less mosquitoes were collected in treated houses compared to control ones, indicating that these two LLINs provided, in addition to the mosquito mortality, a deterrent effect [36] which enhances their expected prevention capacity. However, this study needs to be performed in a high resistance area, such as the Lena village where *An. gambiae *populations are dominated by the S form (more than 95%) with 90% of *kdr *gene [11]: this will permit a more precise evaluation of Permanet^® ^efficacy in an area with pyrethroid resistance.

## Authors' contributions

DKR participated to the study design, undertook the field study, analysed data and wrote the paper. DA participated in the study design, the data analysis and the manuscript drafting. BT participated to the data analysis and interpretation, the drafting and the revision of the paper. TPL participated to study design. GTR and OJB are administrative authorities who facilitated the implementation of the study. SO designed the study, participated to the analysis of data and the drafting of the manuscript.

**Table 3:** Comparison of the global mortality rate and the mortality rates following the mosquito collection place in the two LLINs -equipped houses (percentages are noted in *italic*)

Mosquito status	Mosquito collection place	Olyset^® ^house			Permanet^® ^house			Control house		
			
		Ag	Af	Cq	Ag	Af	Cq	Ag	Af	Cq
	Above LLIN	128	2	2	267	22	10	-	-	-
Dead	Inside LLIN	107	3	5	-	-	-	-	-	-
	Resting indoor	10	0	3	3	0	0	-	-	-
				
		**260**			**302**			**0**		
		***35.3***			***37.5***					

Alive	Above LLIN	0	0	0	0	0	0	0	0	0
	Inside LLIN	0	0	0	0	0	0	0	0	0
	Resting indoor	398	27	66	363	41	102	8,350	110	618
				
		**478**			**503**			**9,808**		

*significantly different, *P*<0.05 Ag = *Anopheles gambiae*; Af = *Anopheles funestus*; Cq = *Culex quinquefasciatus*
